# Mechanism of RCD and the Role of Different Death Signaling Pathways in Cancer

**DOI:** 10.3390/biomedicines13081880

**Published:** 2025-08-02

**Authors:** Jianming Zhou, Ruotong Huang, Maidinai Aimaiti, Qingyu Zhou, Xiang Wu, Jiajun Zhu, Xiangyi Ma, Ke Qian, Qi Zhou, Lianlong Hu, Xiaoyi Yang, Yiting Tang, Yong Lin, Shuying Chen

**Affiliations:** 1 Department of Laboratory Medicine, Huashan Hospital, Fudan University, Shanghai 200040, China; 21301050096@m.fudan.edu.cn (J.Z.); 20301050135@fudan.edu.cn (R.H.); 22301050275@m.fudan.edu.cn (M.A.); kun.kevin@outlook.com (L.H.); 2 Shanghai Medical College, Fudan University, Shanghai 200032, China; 23211340002@m.fudan.edu.cn (Q.Z.); 20301020029@fudan.edu.cn (X.W.); 20301050174@fudan.edu.cn (J.Z.); 21307130347@m.fudan.edu.cn (K.Q.); eriny_0828@163.com (X.Y.); 3 Department of Clinical Laboratory, Central Laboratory, Jing’an District Center Hospital of Shanghai, Fudan University, Shanghai 200040, China; mxyqianqian@126.com; 4 Department of Laboratory Medicine, Shanghai Fifth People’s Hospital, Fudan University, Shanghai 200240, China

**Keywords:** regulated cell death (RCD), autophagy, apoptosis, necroptosis, pyroptosis, ferroptosis

## Abstract

Cancer remains a significant global health challenge, with China being particularly affected because of its large population. Regulated cell death (RCD) mechanisms, including autophagy, apoptosis, necroptosis, pyroptosis, and ferroptosis, play complex roles in cancer development and progression. This review explores the dual roles of autophagy and apoptosis in cancer, highlighting their tumor-suppressive and tumor-promoting functions. Autophagy can maintain genomic stability, induce apoptosis, and suppress protumor inflammation, but it may also support tumor cell survival and drug resistance. Apoptosis, while primarily tumor-suppressive, can paradoxically promote cancer progression in certain contexts. Other RCD mechanisms, such as necroptosis, pyroptosis, and ferroptosis, also exhibit dual roles in cancer, influencing tumor growth, metastasis, and immune responses. Understanding these mechanisms is crucial for developing targeted cancer therapies. This review provides insights into the intricate interplay between RCD mechanisms and cancer, emphasizing the need for context-dependent therapeutic strategies.

## 1. Introduction

Cancer remains one of the most pressing global health challenges, with its burden continuing to rise worldwide. On 2 February 2024, the International Agency for Research on Cancer (IARC) of the World Health Organization (WHO) once again underscored the escalating global cancer burden, emphasizing the urgent need for international intervention. According to recent data, the number of new cancer cases worldwide in 2022 reached 20 million, with 9.7 million cancer-related deaths. An estimated 53.5 million individuals survived at least five years following their cancer diagnosis. Alarmingly, approximately one in five people will develop cancer in their lifetime, with about one in nine men and one in twelve women succumbing to the disease.

As the world’s most populous country, China plays a significant role in the global cancer landscape. The country ranks first in both new cancer cases and cancer-related deaths, making cancer one of the leading causes of mortality in China. Between 1990 and 2019, cancer-related deaths in China surged by 87% [1]. On 17 December 2024, the IARC reported that China continues to lead the world in cancer incidence and mortality. Across Asia, 49.2% of all new cancer cases and 56.1% of cancer-related deaths have been recorded, with China being a major contributor to these statistics [2].

Regulated cell death (RCD) is a biological process that ensures tissue homeostasis by eliminating dysfunctional, damaged, or harmful cells. While RCD is essential for normal cellular function, it can also be triggered by injury or disease, leading to pathological cell death. In the context of cancer, growing evidence suggests a complex relationship between cell death and tumorigenesis. Understanding the mechanisms underlying different forms of RCD is crucial for developing effective therapeutic strategies [3]. Traditionally, RCD has been classified into three main types based on morphological characteristics: apoptosis, autophagic cell death, and necrosis. While this classification provided a foundational framework for early research, the discovery of novel cell death modalities has highlighted its limitations [4]. However, given the extensive research on traditional cell death mechanisms and the relatively limited understanding of emerging forms, this review primarily focuses on autophagy, apoptosis, necroptosis, pyroptosis, and ferroptosis as the key types of RCD.

The rising cancer burden underscores the urgent need for improved research, prevention, and treatment strategies. Understanding the molecular mechanisms driving cancer progression can inform the development of targeted therapeutic approaches. This review aims to explore the dual role of RCD in cancer development, offering insights into the intricate interplay between these processes and tumor progression.

## 2. Diversity and Pathway of RCD

Regulated cell death (RCD) encompasses a variety of mechanisms that ensure the elimination of dysfunctional or harmful cells. While apoptosis, necrosis, and other forms of RCD have been extensively studied, autophagic cell death remains a complex and sometimes ambiguous concept. Autophagic cell death is a form of regulated cell death that involves the process of autophagy, where cells degrade their own components through the formation of autophagosomes and subsequent fusion with lysosomes. This process can be both a survival mechanism and a form of cell death, depending on the context.

**Figure 1:**
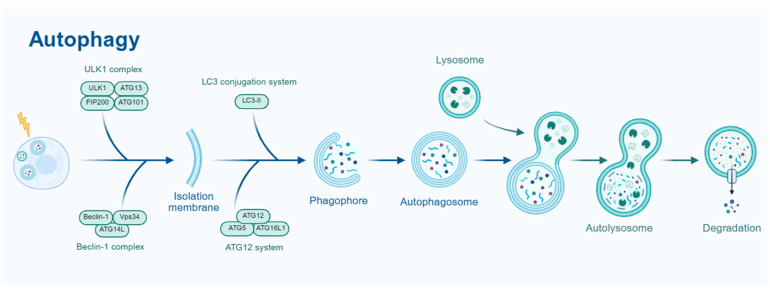
Schematic representation of the autophagy process. The ULK1 complex (ULK1, FIP200) initiates the process, followed by the LC3 conjugation system (LC3-I, ATG12-ATG5-ATG16L) for phagophore formation. The phagophore matures into an autophagosome, which fuses with a lysosome to form an autolysosome for degradation. The Beclin-1 (Vps34) complex and ATG12 system also contribute to autophagosome formation.

Autophagy is a highly conserved cellular degradation process that utilizes lysosomes to remove damaged organelles, misfolded proteins, and intracellular pathogens, thereby maintaining cellular homeostasis and enabling adaptation to environmental changes. Based on its mechanistic differences, autophagy can be categorized into macroautophagy, microautophagy, and chaperone-mediated autophagy (CMA) [5]. Among these, macroautophagy is the most extensively studied form, playing a crucial role in cellular survival, cancer, neurodegenerative diseases, and aging. Therefore, this review primarily focuses on macroautophagy. As shown in Figure 1, the autophagy process involves several key stages, including initiation, nucleation, elongation, maturation, and degradation.

The process of macroautophagy generally consists of five key stages: initiation, nucleation, elongation, maturation, and degradation [6]. Under conditions of nutrient deprivation, oxidative stress, and other cellular stressors, autophagy is initiated by the ULK1 complex (ULK1-ATG13-FIP200-ATG101) [7] and promoted by the Beclin-1 complex (Beclin-1-Vps34-ATG14L) [8], which facilitates autophagosome formation. Subsequently, with the involvement of the ATG12-ATG5-ATG16L1 and LC3 conjugation systems, the autophagosome membrane gradually expands. Eventually, the autophagosome fuses with the lysosome to form an autolysosome, where lysosomal hydrolases degrade the internal substrates. The resulting degradation products are then recycled by the cell, contributing to intracellular material homeostasis [9].

The regulation of autophagy involves multiple signaling pathways, with mTOR, AMPK, p53, and PI3K/AKT being key regulators [10]. Through complex interactions, these pathways precisely control the activation and inhibition of autophagy, playing vital roles in cellular metabolism, stress responses, and disease progression. A deeper understanding of these regulatory mechanisms will provide valuable insights into the physiological and pathological functions of autophagy and offer potential therapeutic strategies for related diseases. The occurrence of autophagy is tightly regulated by multiple signaling pathways that enable cells to adapt to metabolic demands and environmental changes. Among them, the mTOR (mechanistic target of rapamycin) signaling pathway serves as the primary negative regulator of autophagy [11]. Under nutrient-rich conditions, mTORC1 inhibits autophagy initiation by phosphorylating ULK1 (Unc-51-like kinase 1) and ATG13, while also suppressing autophagy-related gene expression by inhibiting TFEB (transcription factor EB), a key regulator of lysosomal biogenesis [12]. However, during nutrient deprivation or energy crisis, mTORC1 is inactivated, relieving its suppression and thereby promoting autophagy initiation [13]. In contrast, the AMPK (AMP-activated protein kinase) signaling pathway is a critical positive regulator of autophagy, activated in response to low cellular energy levels. AMPK enhances autophagy by directly phosphorylating ULK1 and inhibiting mTORC1 via phosphorylation of TSC2 (Tuberous Sclerosis Complex 2) or RAPTOR (a key subunit of the mTORC1 complex), further amplifying autophagic activity [14]. Additionally, the p53 signaling pathway plays a dual role in autophagy regulation: nuclear p53 promotes autophagy by activating DRAM (damage-regulated autophagy modulator) and ATG gene expression, whereas cytoplasmic p53 inhibits autophagy by binding to Beclin-1. This regulatory balance is influenced by cellular stress and environmental conditions [15].

Beyond these pathways, the PI3K/AKT signaling pathway primarily inhibits autophagy by activating mTORC1. Type I PI3K is stimulated by growth factors (such as IGF-1 and EGF), leading to AKT activation, which inhibits the TSC1/TSC2 complex, ultimately stimulating mTORC1 and suppressing autophagy [16]. In contrast, type III PI3K (Vps34) promotes autophagosome formation by forming a complex with Beclin-1. The function of Beclin-1 is modulated by various proteins; for instance, Bcl-2 binds to Beclin-1 to suppress autophagy, while JNK-mediated phosphorylation of Bcl-2 disrupts this interaction, thereby enhancing autophagy [17]. Furthermore, HIF-1α (hypoxia-inducible factor 1α) induces BNIP3 and BNIP3L expression under hypoxic conditions, promoting mitophagy [18], whereas FoxO transcription factors activate ATG genes to facilitate autophagy [19,20]. These signaling pathways are intricately interconnected, collectively regulating autophagy activation and playing vital roles in maintaining cellular homeostasis, stress adaptation, and disease progression.

**Figure 2:**
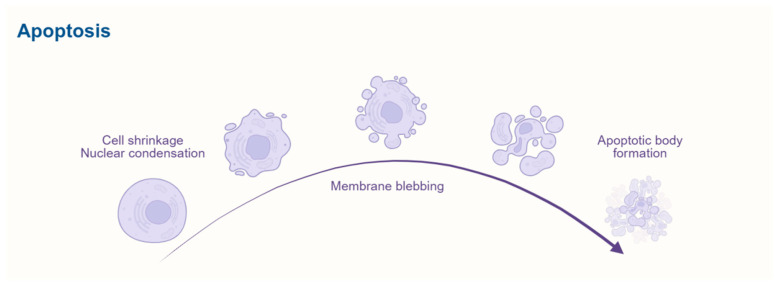
Schematic representation of the apoptosis process. The diagram illustrates key stages of apoptosis, starting with cell shrinkage and nuclear condensation, followed by membrane blebbing, and culminating in the formation of apoptotic bodies. This process is a critical mechanism of programmed cell death, essential for maintaining tissue homeostasis and eliminating damaged cells.

Apoptosis is a highly conserved form of programmed cell death (PCD), compared with autophagy, that plays a crucial role in individual development, tissue homeostasis, and disease progression. Unlike necrosis, apoptosis is tightly regulated by genetic mechanisms and exhibits distinct biological characteristics, including cell shrinkage, nuclear condensation, chromatin condensation, membrane blebbing, and the formation of apoptotic bodies [21]. Notably, apoptosis does not trigger an inflammatory response, making it essential for maintaining cellular and physiological balance [22]. Dysregulation of apoptosis is closely linked to various diseases, including cancer, neurodegenerative disorders, and autoimmune diseases. Therefore, investigating the regulatory mechanisms of apoptosis is of great significance for elucidating its physiological and pathological roles and developing targeted therapeutic strategies. Figure 2 illustrates the key stages of apoptosis, starting with cell shrinkage and nuclear condensation, followed by membrane blebbing, and culminating in the formation of apoptotic bodies.

**Figure 3:**
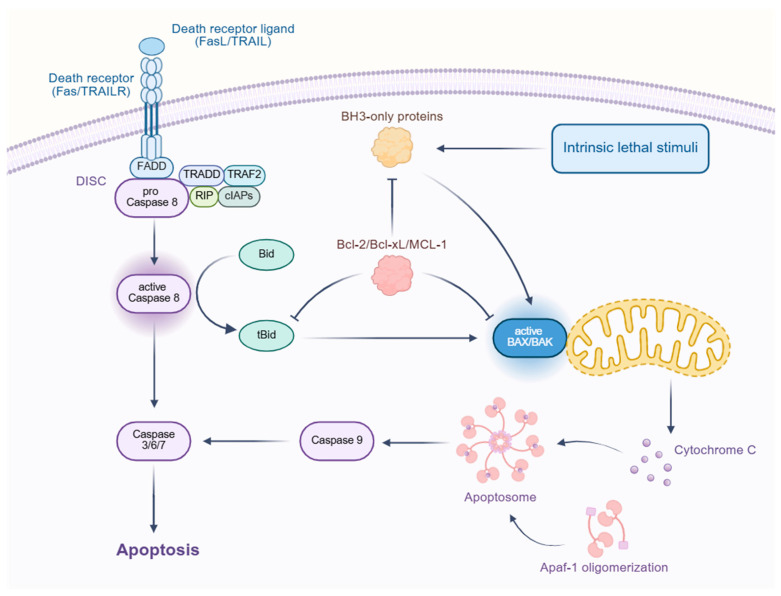
Schematic representation of the apoptotic signaling pathways. The diagram illustrates the extrinsic pathway initiated by death receptor ligands (FasL/TRAIL) binding to death receptors (Fas/TRAILR), leading to the formation of the death-induced signaling complex (DISC). This complex activates caspase-8, which, in turn, activates caspases 3/6/7, leading to apoptosis. The intrinsic pathway is triggered by intrinsic lethal stimuli, activating BH3-only proteins that disrupt the Bcl-2/Bcl-xL/MCL-1 complex, leading to the activation of BAX/BAK and the release of cytochrome C from the mitochondria. Cytochrome C then forms the apoptosome, which activates caspase-9 and, subsequently, caspases 3/6/7, culminating in apoptosis. The diagram also shows the interaction between the extrinsic and intrinsic pathways through Bid.

The regulation of apoptosis involves multiple signaling pathways, including the extrinsic (death receptor) pathway, the intrinsic (mitochondrial) pathway, endoplasmic reticulum (ER) stress signaling, and key regulatory networks, such as the p53, PI3K/AKT, and NF-κB pathways. These pathways interact intricately, collectively determining cell survival or death [23]. The apoptotic signaling pathways, as depicted in Figure 3, include the extrinsic (death receptor) pathway and the intrinsic (mitochondrial) pathway, which interact to determine cell fate.

The extrinsic apoptotic pathway is initiated by death receptors, such as Fas, TNFR1, and TRAIL receptors. Upon binding with their respective ligands (FasL, TNF-α, TRAIL), these receptors undergo trimerization and recruit Fas-Associated Death Domain (FADD) protein, forming the death-inducing signaling complex (DISC). DISC subsequently activates caspase-8 or caspase-10, which, in turn, triggers the caspase cascade, ultimately leading to apoptosis. Additionally, caspase-8 can cleave the BH3-only protein Bid, thereby activating the intrinsic mitochondrial pathway and amplifying the apoptotic signal [23,24].

The intrinsic apoptotic pathway is primarily regulated by mitochondria, with the Bcl-2 family of proteins playing a central role [25]. Under cellular stress, pro-apoptotic proteins, such as Bax and Bak, undergo conformational changes, forming pores in the mitochondrial outer membrane. This leads to the release of cytochrome c, which then binds to Apaf-1, forming the apoptosome. The apoptosome activates caspase-9, which subsequently triggers the activation of caspase-3, executing the apoptotic program. In contrast, anti-apoptotic proteins, such as Bcl-2 and Bcl-xL, inhibit Bax/Bak activation, preventing mitochondrial outer membrane permeabilization and suppressing apoptosis [26]. Furthermore, BH3-only proteins (e.g., Bid, Bim, Puma, and Noxa) can enhance apoptosis by either directly activating Bax/Bak or antagonizing Bcl-2 [27].

In addition to the classical extrinsic and intrinsic pathways, several other signaling pathways play essential roles in apoptosis regulation. The tumor suppressor protein p53 is activated in response to cellular stress, particularly DNA damage. It promotes apoptosis by upregulating the expression of pro-apoptotic proteins, such as Bax, Puma, and Noxa, while simultaneously inhibiting anti-apoptotic proteins, like Bcl-2 and Bcl-xL [28]. Additionally, p53 can enhance apoptosis through upregulation of death receptors, such as Fas and DR5, further reinforcing the apoptotic response [29]. ER stress triggers apoptosis via multiple signaling axes, including the PERK-eIF2α-CHOP, IRE1-ASK1-JNK, and ATF6 pathways. CHOP (C/EBP homologous protein) plays a crucial role in promoting apoptosis by suppressing Bcl-2 and facilitating Bax/Bak activation. Meanwhile, JNK signaling induces the expression of pro-apoptotic proteins, such as Bim and Puma, further promoting apoptosis [30].

The PI3K/AKT pathway plays a pivotal role in suppressing apoptosis and enhancing cell survival. It inhibits apoptosis through multiple mechanisms, including the phosphorylation of caspase-9, the upregulation of anti-apoptotic proteins (such as Bcl-2 and Bcl-xL), and the inhibition of the pro-apoptotic protein Bad, thereby contributing to cellular resistance against apoptotic stimuli [31].

The NF-κB pathway primarily functions to inhibit apoptosis and promote cell survival by inducing the expression of anti-apoptotic proteins, such as Bcl-2, and inhibitor of apoptosis proteins (IAPs). Additionally, NF-κB counteracts p53-mediated apoptosis, thereby sustaining cell viability in various cellular contexts. In certain cases, NF-κB activation can contribute to cancer cell survival by blocking apoptotic pathways [32].

**Figure 4:**
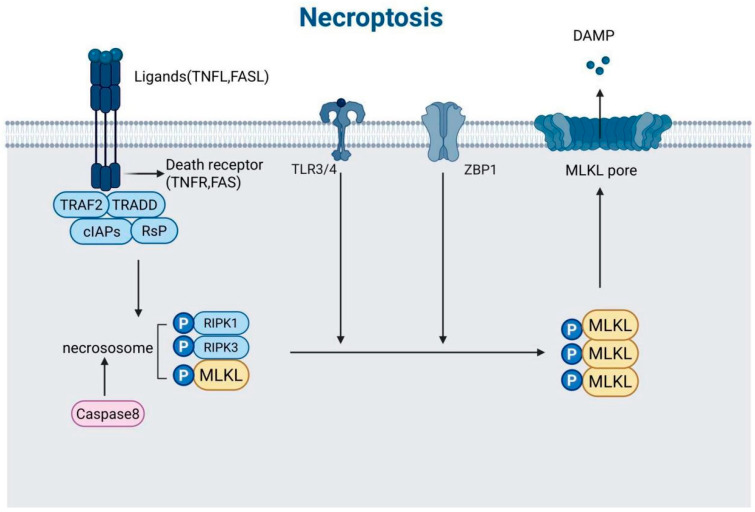
Schematic representation of the necroptosis signaling pathways. The diagram illustrates the initiation of necroptosis by ligands (TNF, FASL) binding to their respective death receptors (TNFR, FAS). This interaction leads to the formation of a complex involving TRAF2, TRADD, cIAPs, and RIPK1. Caspase-8 activation is shown as an alternative pathway that can lead to the formation of the necrosome, which consists of RIPK1, RIPK3, and MLKL. The phosphorylation of MLKL by RIPK3 results in the formation of MLKL pores in the cell membrane, leading to the release of damage-associated molecular patterns (DAMPs) and cell death. The diagram also shows the involvement of ZBP1 and TLR3/4 in necroptosis, indicating multiple pathways that can trigger this form of cell death.

Necroptosis expresses a genetically programmed form of necrotic cell death that is precisely regulated by specific signaling cascades, playing pivotal roles in inflammatory responses, host defense mechanisms, and disease pathogenesis [33]. Distinct from apoptosis, this lytic cell death modality operates independently of caspase activation, instead employing a well-characterized molecular pathway involving receptor-interacting protein kinases 1 and 3 (RIPK1/RIPK3) and mixed lineage kinase domain-like protein (MLKL) [34]. The execution phase culminates in plasma membrane rupture and the subsequent release of damage-associated molecular patterns (DAMPs), including high-mobility group box 1 (HMGB1) and adenosine triphosphate (ATP), which potently stimulate inflammatory responses [35]. As illustrated in Figure 4, necroptosis is initiated by ligands binding to their respective death receptors, leading to the formation of the necrosome complex and the phosphorylation of MLKL.

The necroptotic pathway can be initiated through two principal mechanisms. The canonical pathway is triggered by tumor necrosis factor receptor (TNFR) superfamily activation, wherein TNF-α binding to TNFR1, coupled with caspase-8 inhibition (as occurs during viral infection or pharmacological intervention), facilitates RHIM domain-mediated interaction between RIPK1 and RIPK3 to form the necrosome complex. This molecular platform then phosphorylates MLKL, inducing its oligomerization and subsequent membrane insertion to form permeable pores, ultimately leading to osmotic cell lysis [35]. Alternatively, noncanonical pathways may directly activate the RIPK3-MLKL axis through Toll-like receptors (TLR3/4) or Z-DNA binding protein 1 (ZBP1), particularly during viral RNA sensing or endoplasmic reticulum stress responses [36].

The core regulatory machinery of necroptosis comprises three critical components: RIPK1 serves as a molecular switch whose phosphorylation status (e.g., at Ser166) and ubiquitination pattern determine cellular fate, RIPK3 phosphorylates MLKL at Thr357/Ser358 residues through its kinase activity, and MLKL undergoes conformational changes that expose its N-terminal four-helix bundle domain, thereby compromising membrane integrity [34]. This signaling cascade is further modulated by various cellular parameters, including caspase-8 activity, metabolic status (notably, RIPK3-dependent glycolytic flux), and oxidative stress levels [37].

Under physiological conditions, necroptosis contributes to antiviral defense mechanisms (as exemplified by herpesvirus infections) and maintenance of tissue homeostasis (particularly in intestinal epithelial turnover). Pathologically, its dysregulation has been implicated in numerous disease states, including ischemia–reperfusion injury (myocardial infarction and stroke), neurodegenerative disorders (Alzheimer’s disease and amyotrophic lateral sclerosis), and chronic inflammatory conditions (inflammatory bowel disease and psoriasis) [38]. Consequently, pharmacological targeting of necroptotic components, such as RIPK1 inhibitor Necrostatin-1 and RIPK3 antagonist GSK872, has emerged as a promising therapeutic strategy [39,40].

The necroptotic regulatory network exhibits extensive crosstalk with other programmed cell death pathways. Caspase-8 demonstrates dual functionality, both suppressing necroptosis through RIPK1/RIPK3 cleavage and executing apoptotic signaling. Furthermore, synergistic interactions between MLKL and gasdermin D (GSDMD), the pyroptosis effector protein, can amplify inflammatory responses [41].

**Figure 5:**
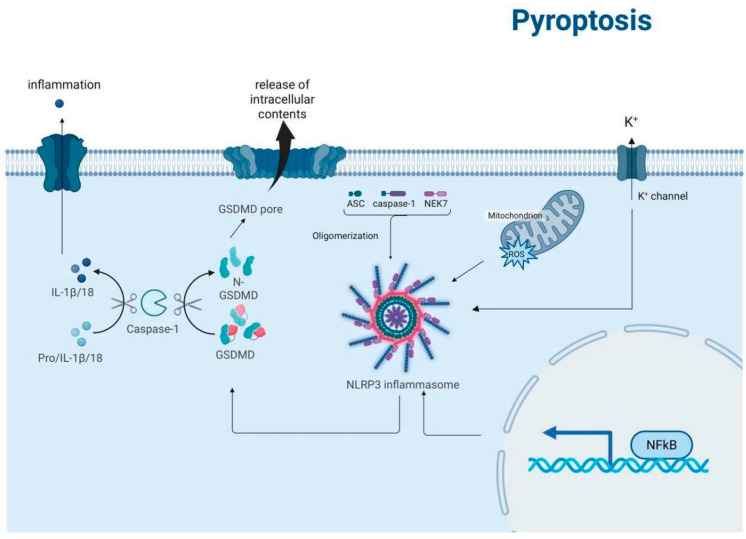
Schematic representation of the pyroptosis signaling pathways. The diagram illustrates the activation of pyroptosis by inflammation, leading to the processing of pro-IL-1β/18 into mature IL-1β/18 by caspase-1. This process involves the formation of the NLRP3 inflammasome, which is activated by ASC, caspase-1, and NEK7 oligomerization. The activation of gasdermin D (GSDMD) by caspase-1 results in the formation of GSDMD pores in the cell membrane, leading to the release of intracellular contents and the influx of potassium ions (K+) through K+ channels. Additionally, the diagram shows the role of mitochondria and reactive oxygen species (ROS) in this process, as well as the activation of NF-κB, which is involved in the regulation of inflammatory responses.

Pyroptosis represents a lytic and inflammatory form of programmed cell death characterized by Gasdermin family protein-mediated plasma membrane pore formation, leading to the release of proinflammatory cytokines and cellular contents [42]. This evolutionarily conserved process serves dual physiological roles as a crucial defense mechanism against intracellular pathogens and a key regulator of immune homeostasis. Figure 5 shows the activation of pyroptosis by inflammation, leading to the processing of pro-IL-1β/18 into mature IL-1β/18 by caspase-1, and the formation of GSDMD pores in the cell membrane.

Currently, the most extensively characterized pathway is the canonical pyroptosis pathway, which is mediated by inflammasome activation and caspase-1-dependent cleavage of gasdermin D (GSDMD) [43]. This pathway involves four distinct molecular events: (1) danger signal perception, (2) inflammasome assembly, (3) caspase activation, and (4) gasdermin execution [44]. Upon detection of pathogen-associated molecular patterns (PAMPs) or damage-associated molecular patterns (DAMPs) through pattern recognition receptors (PRRs), cells assemble multiprotein inflammasome complexes, such as NLRP3-ASC–pro-caspase-1. Subsequent caspase-1 activation mediates the proteolytic cleavage of both pro-IL-1β/pro-IL-18 precursors and GSDMD. The liberated N-terminal fragments of GSDMD undergo oligomerization and insert into the plasma membrane to form functional pores, resulting in cytokine secretion, osmotic imbalance, and eventual plasma membrane rupture [45].

The pyroptotic cascade is precisely regulated by an intricate network of signaling pathways, with inflammasomes, caspases, and gasdermin proteins constituting the core regulatory machinery. The NLRP3 inflammasome serves as a critical molecular hub that integrates diverse danger signals, including extracellular ATP, crystalline substances, and microbial components. Upon activation, NLRP3 recruits the adaptor protein ASC and pro-caspase-1 to form functional inflammasomes. In parallel, caspase-4/5/11 mediate the noncanonical pathway through direct sensing of intracellular LPS and subsequent GSDMD cleavage. The gasdermin protein family, particularly GSDMD and GSDME, functions as terminal effectors whose pore-forming activity is strictly controlled by specific caspase-mediated proteolytic cleavage [45,46].

The regulation of pyroptosis involves complex crosstalk among multiple signaling networks. The NF-κB pathway provides priming signals for pyroptosis by upregulating NLRP3 and pro-IL-1β expression [47]. Potassium efflux and mitochondrial reactive oxygen species (mtROS) generation represent two critical checkpoints for NLRP3 inflammasome activation [48]. Furthermore, autophagy negatively regulates pyroptosis by eliminating damaged mitochondria and inflammasome components, while the cGAS-STING pathway potentiates pyroptotic responses to cytosolic DNA [49]. Post-translational modifications, including NLRP3 phosphorylation and ASC ubiquitination, provide additional layers of regulatory control to fine-tune pyroptotic signaling [50].

**Figure 6:**
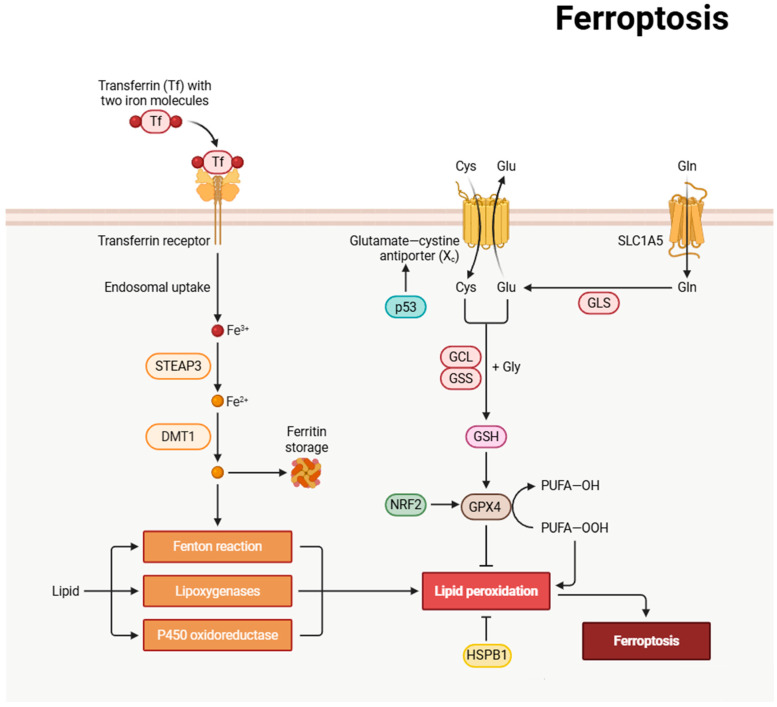
Schematic representation of the ferroptosis signaling pathways. The diagram shows iron uptake via the transferrin receptor, leading to Fenton reactions and lipid peroxidation. The glutamate–cysteine antiporter and System Xc- facilitate cystine uptake for glutathione (GSH) synthesis, which is crucial for GPX4-mediated lipid peroxide detoxification, preventing ferroptosis. The figure highlights the roles of p53 and NRF2 in regulating antioxidant defenses.

Ferroptosis is an iron-dependent form of regulated cell death characterized by the aberrant accumulation of lipid peroxides and disruption of redox homeostasis. Distinct from other cell death modalities, such as apoptosis, necroptosis, and autophagy, ferroptosis plays critical roles in diverse physiological and pathological processes, including tumor suppression, neurodegenerative diseases, and ischemia–reperfusion injury [51]. Based on regulatory mechanisms, ferroptosis can be classified into two main pathways: glutathione peroxidase 4 (GPX4)-dependent [52] and GPX4-independent [53]. The GPX4-dependent pathway, being the most extensively studied, occupies a central position in cellular metabolism, oxidative stress responses, and disease pathogenesis. Figure 6 highlights the roles of iron uptake via the transferrin receptor, leading to Fenton reactions and lipid peroxidation, and the involvement of p53 and NRF2 in regulating antioxidant defenses.

The execution of GPX4-dependent ferroptosis involves a cascade of molecular events. Under conditions of iron overload or oxidative stress, ferroptosis is triggered through dysfunction of the System Xc^−^–glutathione–GPX4 antioxidant axis [54,55]. Specifically, impaired activity of the System Xc^−^ transporter (composed of SLC7A11 and SLC3A2) leads to reduced cystine uptake and consequent glutathione (GSH) depletion. As a pivotal antioxidant enzyme, GPX4 becomes incapacitated in GSH-deficient conditions, failing to adequately reduce lipid peroxides. This results in peroxidative damage to polyunsaturated fatty acids (PUFAs) in plasma membranes and ultimate loss of membrane integrity. The process is further modulated by lipid metabolism enzymes such as ACSL4 and LPCAT3, which facilitate PUFA esterification and membrane localization, thereby providing substrates for lipid peroxidation [56].

Ferroptosis is precisely regulated by multiple signaling pathways that adapt to cellular metabolic states and environmental stresses. The GPX4–System Xc^−^–GSH axis constitutes the core regulatory system of ferroptosis. Under physiological conditions, the cystine/glutamate antiporter System Xc^−^ maintains intracellular GSH levels, while GPX4 utilizes GSH to reduce lipid peroxides into corresponding alcohols, thereby preventing ferroptosis. However, upon exposure to ferroptosis inducers (e.g., erastin or RSL3), inhibition of System Xc^−^ activity or inactivation of GPX4 function leads to collapse of the antioxidant defense system [57]. Additionally, iron metabolism-related proteins, such as transferrin receptor 1 (TFR1) and ferritin, regulate cellular sensitivity to ferroptosis by modulating intracellular iron levels [58]. Other molecular players, including p53 [59], NRF2 [60], and HSPB1 [61], also participate in the ferroptosis regulatory network.

The p53 signaling pathway exhibits dual roles in ferroptosis regulation. Nuclear p53 can transcriptionally upregulate SLC7A11 expression to inhibit ferroptosis, whereas, under certain stress conditions, p53 may promote ferroptosis by downregulating SLC7A11 or activating prooxidant genes like SAT1 [60]. NRF2, as a master regulator of the oxidative stress response, confers resistance to ferroptosis by orchestrating the expression of various antioxidant genes, including SLC7A11 and GPX4. HSPB1 protects against ferroptosis by stabilizing the cytoskeleton and suppressing lipid peroxidation [60]. Furthermore, ferroptosis demonstrates intricate crosstalk with other cell death pathways. For instance, autophagy may promote ferroptosis through ferritin degradation (ferritinophagy) [62], while anti-apoptotic Bcl-2 family proteins may influence ferroptosis progression by modulating mitochondrial function [63].

In addition to the aforementioned categories of regulated cell death (RCD), several other forms have been identified, including autophagy-dependent cell death, such as PARP-1-dependent cell death (parthanatos), NETosis (unique to neutrophils), and entosis (cell death by neighboring cell engulfment), as well as newly discovered modalities, such as cuproptosis and disulfidptosis [3]. However, given that the current research on these cell death types remains relatively preliminary and superficial, they will not be elaborated upon in this review.

## 3. Cancer Regulation with RCD

### 3.1. Autophagy

Autophagy exhibits a remarkable dual role in cancer, capable of both suppressing tumorigenesis and promoting cancer progression, with its specific effects depending on the tumor stage, genetic background, and microenvironmental conditions [64]. Below, we analyze its tumor-suppressive and tumor-promoting functions in detail from the perspectives of molecular mechanisms, functional regulation, and clinical translation.

#### 3.1.1. Tumor-Suppressive Mechanisms of Autophagy

**a.**  **Clearance of Damaged Mitochondria (Mitophagy)**

Mitophagy, a critical form of selective autophagy, plays a key role in maintaining cellular homeostasis and suppressing tumorigenesis [65]. When cells are exposed to radiation, chemotherapeutic drugs, or oxidative stress, mitochondrial function is impaired, leading to a decline in membrane potential (ΔΨm), dysfunction of the electron transport chain, and excessive accumulation of reactive oxygen species (ROS). These excess ROS can attack nuclear DNA, induce gene mutations, and promote genomic instability, thereby increasing the risk of tumorigenesis [66].

At the molecular level, the PINK1/Parkin pathway is the core regulator of mitophagy. In healthy mitochondria, the PINK1 protein is rapidly degraded via a membrane potential-dependent proteasomal pathway [67]. However, when mitochondrial damage causes loss of membrane potential, PINK1 stably accumulates on the outer mitochondrial membrane (OMM) [68]. PINK1 then phosphorylates and recruits Parkin, which translocates to damaged mitochondria. Parkin ubiquitinates OMM proteins (e.g., VDAC, MFN2) to generate an “eat-me” signal. Autophagy receptor proteins (e.g., p62/SQSTM1, OPTN) recognize these ubiquitin tags, mediating autophagosome formation and subsequent lysosomal degradation [69].

Studies indicate that mitophagy plays a significant role in tumor suppression. For example, in PTEN-deficient tumor cells, hyperactivation of the PI3K/AKT/mTOR pathway increases mitochondrial biogenesis and ROS accumulation. These cells heavily rely on mitophagy to eliminate dysfunctional mitochondria, thereby preventing ROS-induced cell death [70]. This suggests that mitophagy suppresses tumorigenesis in specific genetic contexts.

However, some cancer cells may upregulate mitophagy to sustain survival and develop therapy resistance. For instance, in breast cancer, tumor cells can activate the PINK1/Parkin pathway to clear chemotherapy-damaged mitochondria, leading to drug resistance. Inhibiting this pathway significantly enhances chemosensitivity. This dual role makes targeted modulation of mitophagy a potential anticancer strategy [71].

**b.**  **Degradation of Misfolded Proteins**

As a highly conserved intracellular degradation system, autophagy selectively eliminates various oncoproteins, exerting potent antitumor effects [72]. In cancer cells, the homeostasis of mutant p53 is dysregulated: its classical proteasomal degradation is often impaired, shifting its clearance to macroautophagy and chaperone-mediated autophagy (CMA). Under nutrient deprivation, glucose starvation induces autophagy and enhances mutant p53 degradation via acetylation. Notably, even when both autophagy and proteasome functions are inhibited, CMA can still effectively degrade mutant p53 in non-proliferating tumor cells, highlighting the complementary roles of different autophagy pathways in tumor suppression [73].

Additionally, the autophagy regulator Beclin-1 influences p53 stability through direct interaction. The BH3 domain of Beclin-1 binds p53, facilitating its ubiquitin–proteasome system (UPS)-dependent degradation. Beclin-1 degradation not only reduces overall autophagy but also affects p53 stability, influencing tumor cell fate [74].

In oncoprotein degradation, the autophagy receptor p62 (SQSTM1) serves as a central player. As a multifunctional scaffold protein, p62 is often overexpressed in cancers. Its protumor effects are evident in autophagy-deficient cells, where accumulated p62 activates the NRF2 antioxidant pathway and mTORC1/c-Myc proliferative network, promoting hepatocellular carcinoma (HCC) progression. Experimental evidence shows that in diethylnitrosamine (DEN)-induced HCC, p62 accumulation stabilizes NRF2 and activates mTORC1, accelerating tumorigenesis. Conversely, autophagy-mediated p62 degradation blocks these protumor signals, suppressing HCC [75].

Other oncoproteins, such as PML-RARA (a marker of acute promyelocytic leukemia) and BCR-ABL1 (chronic myeloid leukemia), are also degraded via autophagy. Clinical drugs like arsenic trioxide (ATO) enhance autophagy to promote p62-dependent degradation of these proteins [76]. Notably, p62 physically interacts with BCR-ABL1, targeting it for autophagic degradation—a key insight into autophagy’s antileukemic mechanisms.

#### 3.1.2. Induction of Apoptosis and Senescence

**a.**  **p53-Dependent Pathways**

Upon DNA damage, p53 upregulates autophagy-related genes [77] to degrade inhibitors of pro-apoptotic factors [78], indirectly activating apoptosis. As a tumor suppressor, p53 exerts dual roles in autophagy regulation based on its subcellular localization. Cytoplasmic p53 inhibits autophagy via mTORC1 activation, while nuclear p53 activates autophagy via AMPK/mTOR. Under genotoxic stress, p53 transcriptionally upregulates ULK1/2, promoting autophagic cell death [14].

The RB-E2F pathway also regulates autophagy. Under oxidative stress, RB1 inhibits E2F transcription factors (e.g., E2F1), which otherwise induce autophagy genes (ATG1, ATG5, LC3). Autophagy, in turn, modulates RB-E2F activity, forming a dynamic balance that determines cell fate [79].

**b.**  **Autophagic Cell Death**

Excessive autophagy can lead to cell death via over-degradation of critical components. In breast cancer, high autophagic flux induces Beclin-1-dependent death, demonstrating its tumor-suppressive role [80]. Beclin-1’s antitumor effects are observed in various cancers, including laryngeal squamous cell carcinoma, where its overexpression inhibits proliferation and induces apoptosis [81].

Beclin-1 interacts with UVRAG to activate the BECLIN1-PI3KC3 complex, suppressing colon cancer growth [82]. BCL-2 inhibits autophagy by binding Beclin-1, but JNK1 phosphorylates BCL-2 to disrupt this interaction, activating starvation-induced autophagy. DAPK1 phosphorylates Beclin-1 to promote autophagy and inhibit tumor growth. ULK1/2, key autophagy initiators, are downregulated in gliomas, suggesting that their upregulation may induce autophagic death [17].

**c.**  **Cell Cycle Regulation**

Autophagy inhibits tumor proliferation by inducing cell cycle arrest. In pancreatic cancer, enhanced autophagy causes G2/M arrest and death [83]. Metformin-induced autophagy blocks multiple myeloma cells in G0/G1 via AMPK-mTORC1 [84]. Autophagy also contributes to oncogene-induced senescence (OIS) [85]. In breast cancer, autophagy and senescence jointly suppress self-renewal after doxorubicin treatment. Silencing ATG5 or ATG7 inhibits OIS, while ULK3 overexpression reduces fibroblast proliferation [86].

#### 3.1.3. Suppression of Protumor Inflammation

**a.**  **Degradation of Inflammasomes**

Autophagy inhibits the “chronic inflammation–fibrosis–cancer” axis by degrading NLRP3 inflammasomes and damaged lysosomes, blocking IL-1β release [86]. This is critical in hepatitis-to-HCC progression. Autophagy clears proinflammatory cytokines and damaged mitochondria, preventing infection-related cancers [87].

In viral carcinogenesis (e.g., HBV, HPV), xenophagy (pathogen-selective autophagy) acts as the first defense. Autophagy deficiency in HBV-associated HCC promotes inflammasome activation and IL-1β release, accelerating fibrosis and tumorigenesis [87]. Similar mechanisms exist in H. pylori-induced gastric cancer and HPV-linked cervical cancer [88,89].

**b.**  **Immunomodulation of the Tumor Microenvironment**

Autophagy-deficient macrophages secrete excess IL-6 and TNF-α, activating cancer-associated fibroblasts (CAFs) [90,91]. Normal autophagy suppresses this by clearing damaged mitochondria and inflammasomes [92]. In pathogen-driven cancers, xenophagy eliminates oncogenic viruses (HBV, HPV), but viruses evade clearance by encoding autophagy inhibitors—a potential therapeutic target.

Notably, autophagy has dual roles in cancer stem cells (CSCs). While its loss causes hematopoietic stem cell hyperproliferation, autophagy maintains breast CSC stemness via IL-6 regulation [93]. This cell type-specific duality underscores the context-dependent nature of autophagy in cancer.

#### 3.1.4. Supporting Tumor Cell Survival

**a.**  **Nutrient Recycling**

Metabolic reprogramming is a hallmark of malignant tumors, most notably manifested by the Warburg effect—the preferential use of glycolysis even under oxygen-sufficient conditions [94]. This metabolic shift promotes tumor progression through multiple mechanisms: (1) rapid ATP generation to meet energy demands; (2) provision of biosynthetic precursors to support cell proliferation; and (3) lactate production leading to microenvironment acidification, which activates MMPs to facilitate invasion and metastasis. The PI3K-AKT-mTOR signaling pathway serves as the central hub regulating this process by upregulating transcription factors HIF-1α and c-Myc, thereby promoting expression of key glycolytic enzymes (GLUT1/3 transporters, HK2, PKM2) while inhibiting PDK-mediated pyruvate entry into the TCA cycle [31].

Autophagy exhibits a complex and sophisticated regulatory network in tumor metabolic adaptation. At the cell-autonomous level, autophagy degrades damaged organelles and biomacromolecules through “metabolic recycling,” providing tumor cells with critical metabolic substrates, such as amino acids and fatty acids. Particularly under nutrient-deprived conditions, autophagy activation maintains cancer stem cell properties and promotes chemoresistance [95]. At the tumor microenvironment level, autophagy establishes metabolic symbiosis by regulating the activation state of CAFs: CAFs produce metabolites like alanine through autophagy, which are taken up by tumor cells and converted into α-ketoglutarate in the TCA cycle to support tumor growth [96]. This metabolic coupling is especially prominent in apoptosis-deficient tumors, where autophagy inhibition can lead to metabolic crisis and cell death. Recent studies have also revealed that tumor cells can remodel premetastatic niches through exosome-mediated delivery of autophagy-related miRNAs (e.g., miR-155), preparing favorable metabolic conditions for metastasis formation [97]. These findings not only deepen our understanding of tumor metabolic heterogeneity but also provide theoretical foundations for developing therapeutic strategies targeting the tumor metabolism–autophagy axis.

**a.**  **Resistance to Metabolic Stress**

**Metabolic Stress**

In nutrient-deprived tumor microenvironments, tumor cells reprogram their metabolic networks through the AMPK/mTORC1 signaling axis. When the AMP/ATP ratio increases, AMPK directly initiates autophagy by phosphorylating ULK1 (S317, S555) and Beclin-1 (S91/S94), while simultaneously inhibiting mTORC1 activity through TSC2 [98]. This dual regulation ensures maintenance of basic metabolic demands during energy crises. Studies demonstrate that autophagy-deficient tumor cells show significantly reduced survival rates under glucose deprivation, confirming the crucial role of autophagy in metabolic stress [99]. Notably, different tumor types exhibit varying sensitivities to metabolic stress, with pancreatic cancer displaying particularly strong autophagy dependence [100].

**Hypoxic Stress**

The hypoxic microenvironment in solid tumors activates autophagy through multiple pathways. Mechanistically, hypoxia inhibits PHD2 activity, leading to HIF-1α stabilization, which upregulates BNIP3 expression and dissociates the Beclin-1/BCL-2 complex [101]. Clinical sample analyses reveal significantly elevated expression of autophagy marker LC3B in hypoxic tumor regions, correlating with poor prognosis [102]. Of particular interest, hypoxia-induced autophagy may maintain cancer stem cell properties, providing new perspectives for understanding tumor recurrence. The latest research suggests intermittent hypoxia may promote tumor adaptive evolution through periodic autophagy activation [101].

**Oxidative Stress**

Reactive oxygen species (ROS) accumulation triggers the ATM-LKB1-AMPK cascade while upregulating p62 expression via NF-κB. Autophagy activates the antioxidant defense system through the p62-KEAP1-NRF2 axis, establishing a dynamic equilibrium. This bidirectional regulation shows spatiotemporal specificity: acute oxidative stress induces protective autophagy, while sustained stress may lead to autophagic flux impairment [103]. In KRAS-mutant tumors, moderate autophagy inhibition can induce lethal oxidative damage, but complete blockade may activate compensatory mechanisms, highlighting the need for precise intervention modulation.

In the context of autophagy, ROS function as a double-edged sword. Moderate ROS levels can trigger protective autophagy to maintain cellular homeostasis and safeguard cells from oxidative damage, which is particularly crucial in cancer cells that often have heightened metabolic activity and ROS production. Autophagy helps these cells survive by recycling damaged organelles and macromolecules, providing essential nutrients and energy under stress. For example, in KRAS-mutant tumors, autophagy is vital for cancer cell survival and chemoresistance under oxidative stress [104]. Conversely, excessive ROS can overwhelm the cellular antioxidant defenses, impairing autophagic flux and leading to the accumulation of damaged cellular components. This can cause genomic instability and activate cell death pathways, potentially driving cancer initiation. Sustained oxidative stress may induce DNA damage and mutations, transforming normal cells into cancerous ones. Additionally, ROS-induced damage to mitochondrial DNA can impair mitochondrial function, further increasing ROS production and creatin.

**Endoplasmic Reticulum Stress**

ER stress, an important stressor in the tumor microenvironment caused by calcium homeostasis imbalance, oxidative stress, and protein folding defects, leads to the accumulation of unfolded/misfolded proteins in the ER lumen. To cope, tumor cells activate autophagy through the unfolded protein response (UPR), forming a crucial survival mechanism. Research shows UPR mainly induces autophagy via the PERK-eIF2α-ATF4 signaling axis, promoting LC3-I to LC3-II conversion, a process that plays protective roles in breast cancer radioresistance and tamoxifen treatment [104]. Meanwhile, the IRE1 pathway mediates stress-induced autophagy in neuroblastoma and colorectal cancer, while GRP78 upregulation generally enhances autophagic flux [105]. Notably, this stress adaptation mechanism has dual effects: in cervical cancer, HPV collaborates with the NF-κB pathway to promote cell death through the ER stress–autophagy axis [106], while in colorectal cancer, apatinib-induced protective autophagy leads to acquired resistance [107].

#### 3.1.5. Drug Resistance and Immune Evasion

**a.**  **Chemoresistance**

Chemoresistance remains a major challenge limiting anticancer drug efficacy, involving multiple mechanisms including reduced drug uptake, efflux pump overexpression, impaired drug penetration, abnormal metabolic activation, apoptosis pathway defects, and enhanced DNA repair [108]. Recent studies demonstrate that autophagy plays complex roles in chemoresistance, potentially enhancing certain drugs’ antitumor effects while more commonly mediating resistance development [109]. Various clinical chemotherapeutic agents can induce protective autophagy, including targeted therapies (e.g., gefitinib/erlotinib in lung cancer, imatinib in leukemia), DNA-damaging agents (e.g., temozolomide in glioblastoma, camptothecin in breast cancer), antimetabolites (e.g., 5-FU in colorectal cancer), and endocrine therapies (e.g., tamoxifen/trastuzumab in breast cancer). These drugs activate tumor cell protective autophagy mechanisms by inducing stress conditions like nutrient deprivation, hypoxia, oxidative stress, and DNA damage [110]. Specifically, autophagy delays camptothecin-induced cell death in breast cancer [111] while exerting cytoprotective effects against 5-FU in colorectal and esophageal cancers [112]. Meanwhile, tumor cells also acquire apoptosis resistance through multiple mechanisms, including upregulation of inhibitor of apoptosis proteins (IAPs), NF-κB pathway activation, and BCL-2 family protein expression imbalance [113]. In gastric cancer, for example, long-term scutellarein treatment can effectively reverse tumor cell apoptosis resistance by downregulating MDM2, activating p53 signaling, and subsequently inhibiting IAP expression [114]. Based on these findings, autophagy inhibition has become an important strategy to restore tumor cell chemosensitivity, including using LC3 siRNA combined with imatinib to enhance breast cancer sensitivity to trastuzumab, employing chloroquine (CQ) and hydroxychloroquine (HCQ) to induce apoptotic and necrotic death by blocking autophagosome degradation, and combining autophagy inhibitors with conventional chemotherapy to significantly improve efficacy [115].

**b.**  **Immune Evasion**

Autophagy plays a key role in tumor immune evasion. Research shows autophagy participates in immune escape through multiple mechanisms: at the molecular level, autophagy selectively degrades MHC class I molecules and tumor-specific antigens, significantly reducing tumor cell immunogenicity, while regulating immune checkpoint molecules like PD-L1 to suppress cytotoxic T cell activity [116]. In various solid tumors (e.g., non-small cell lung cancer, melanoma), high autophagy activity shows a significant negative correlation with CD8+ T cell infiltration in the tumor microenvironment [117]. Notably, autophagy can also promote intrinsic resistance to immunotherapy by maintaining cancer stem cell properties. Therapeutically, targeting autophagy pathways has emerged as a novel strategy to enhance cancer immunotherapy efficacy. Preclinical studies confirm that combining autophagy inhibitors (e.g., chloroquine) with PD-1/PD-L1 blockade significantly improves treatment outcomes [118]. Currently, multiple clinical trials are evaluating the safety and efficacy of this combination strategy in advanced cancer patients. However, this field still faces major challenges, including how to precisely regulate autophagy activity to avoid systemic immunosuppression and how to overcome treatment resistance caused by tumor heterogeneity.

### 3.2. Apoptosis

As previously discussed, apoptosis is a form of programmed cell death that can inhibit the occurrence and development of tumors by eliminating malignant or precancerous cells, thereby exerting a cancer-suppressive effect [21]. An increasing number of studies have highlighted the need to pay more attention to the apoptosis paradox. Although the apoptosis mechanism can inhibit tumors by clearing cancerous or precancerous cells, in many high-grade cancers, the level of apoptosis is high, a phenomenon closely related to the aggressiveness and poor prognosis of cancer. It is important to emphasize the significant cancer-suppressive role of apoptosis, which mainly includes the inhibition of the apoptotic process and the alteration of the tumor microenvironment (TME), and even apoptotic cells can directly induce the repair and regeneration of tumor cells [119].

The development of cancer is a process in which cells gradually evolve from a normal physiological state to malignant proliferation under the cumulative effects of a series of genetic mutations and hereditary changes [120]. In this process, the ignorance or resistance of cells to death signals becomes one of the important factors driving their malignant transformation. The weakening or failure of apoptosis mechanisms allows cells to evade normal death programs, thus playing an extremely critical role in the occurrence and development of cancer. As a highly regulated cell death mechanism, apoptosis is originally responsible for clearing damaged or abnormal cells to maintain the dynamic balance of tissue cell numbers. Therefore, any defects in the apoptotic pathway may lead to the accumulation of cancer cells.

#### 3.2.1. Abnormalities in Apoptosis Proteins

p53 Protein—Numerous studies have shown that defects in the p53 gene are associated with over 50% of human cancers. One study found that in melanoma cells, the abnormal expression of some p53 target genes affects apoptosis and cell cycle regulation, leading to p53 activity disorders and promoting cell proliferation [121]. In contrast, the p53 N-terminal deletion mutant (Δ122p53) mouse model showed reduced survival rates, more aggressive tumors, accelerated cell proliferation, decreased apoptosis, and a strong proinflammatory phenotype [122]. In addition, silencing the p53 mutant can reduce the growth of cancer cell colonies because it induces apoptosis [123,124].

Bcl-2 Family Proteins—Apoptosis regulation requires the Bcl-2 family of proteins, which includes both pro- and anti-apoptotic proteins. The regular process of cell apoptosis can be interfered with by an imbalance within this family, which is typified by the overexpression of anti-apoptotic proteins or the underexpression of pro-apoptotic proteins (or both) [125]. In particular, it has been demonstrated that Bcl-2 overexpression prevents prostate cancer cells from passing through apoptosis, prevents TRAIL-induced apoptosis in neuroblastoma, glioblastoma, and breast cancer cells, and gives tumor cells multidrug resistance [126,127]. The bax gene often mutates in microsatellite-unstable colorectal cancer, impairing apoptosis and making the malignancy resistant to treatment. Similar to this, malignant cells in chronic lymphocytic leukemia (CLL) have an anti-apoptotic phenotype that is typified by lower Bax levels and high Bcl-2 levels [128]. This leads to less apoptosis rather than more proliferation. Furthermore, there is a negative correlation between drug-induced apoptosis and the increased Bcl-2/Bax ratio in CLL patients’ B cells [129].

IAPs (Inhibitor of Apoptosis Proteins)—IAP expression is frequently dysregulated in a variety of malignancies. Researchers discovered that chemoresistance and aberrant IAP family expression in pancreatic cancer cells are tightly connected, with cIAP-2 expression being most strongly linked to drug resistance [130]. Furthermore, gliomas exhibit increased Apollon, which results in resistance to camptothecin and cisplatin [131]. Many types of cancer overexpress survivin. Hematopoietic cells that overexpress Survivin are less prone to apoptosis, according to research. Survivin and XIAP overexpression in non-small cell lung carcinomas (NSCLCs) gives the tumors the capacity to withstand a range of apoptosis-inducing stimuli [132].

#### 3.2.2. Abnormalities in Apoptosis Signaling Pathways

Death receptors and their ligands play a central role in regulating the extrinsic apoptosis pathway, but they may also become key nodes for cells to escape apoptosis. Studies have revealed a variety of mechanisms that can lead to escape from the extrinsic apoptosis pathway, such as reduced receptor expression, functional disorders, and weakened death signals, all of which disrupt normal signal transduction and ultimately reduce the rate of cell apoptosis [133]. Specifically, the downregulation of CD95 expression is closely related to treatment resistance in leukemia and neuroblastoma. In addition, reduced membrane expression of death receptors and abnormal expression of decoy receptors have been confirmed to be associated with escape from the apoptosis signaling pathway in various cancers [134,135]. Some studies have further pointed out that during the development of cervical cancer, the loss of Fas and the dysregulation of FasL, DR4, DR5, and TRAIL may disrupt the balance between cell proliferation and apoptosis, thereby promoting the occurrence of cervical cancer [136].

### 3.3. Necroptosis

Necroptosis, as an alternative form of cell death when autophagy and apoptosis fail, has garnered significant attention in the field of tumor biology in recent years. As a regulated form of programmed cell death, the complex interplay between necroptosis and cancer has become a focal point of the current research. Necroptosis plays a dual role in tumor development and progression: it can both inhibit tumor growth and promote tumor progression and metastasis [33]. The specific mechanisms involve three aspects: first, the expression regulation of RIPK3 (Receptor-Interacting Protein Kinase 3) and its central role in the necroptosis signaling pathway [137]; second, the immune regulatory functions of necroptosis in the tumor microenvironment, including its effects on inflammatory responses and immune cell activation [138]; and, finally, the regulatory effects of necroptosis on the process of tumor metastasis [139].

#### 3.3.1. Immune Regulation (RIPK3 Expression)

Immune surveillance is the process by which the immune system identifies and eliminates cancerous or precancerous cells through tumor-specific antigens (TSAs) or tumor-associated antigens (TAAs), thereby preventing these cells from posing a threat to health. This process involves innate and adaptive immune cells and their effector molecules, including dendritic cells (DCs), cytotoxic T cells, M1-type macrophages, natural killer (NK) cells, natural killer T (NKT) cells, and their respective cytokines [140]. RIPK3 plays a key role in regulating cytokine expression in DCs, thereby modulating immune homeostasis through the expression of regulatory cytokines and bridging the innate and adaptive immune systems. Additionally, RIPK3 can regulate NKT cell function by activating mitochondrial phosphoglycerate mutase 5 (PGAM5), promoting NKT cell-mediated antitumor immune responses independently of the necroptosis pathway [141]. Although the role of apoptosis in maintaining central tolerance has been well defined, necroptosis plays a regulatory role in the antigen-induced proliferation of T cells, primarily by eliminating excess T cells to maintain peripheral T cell homeostasis and the survival of T cells after activation. This process is negatively regulated by caspase-8 [142]. Necroptosis occurs in the later stages of T cell proliferation, and the necroptosis signal is significantly enhanced in T cells lacking FADD, indicating that FADD may negatively regulate necroptosis mediated by the T cell receptor. Necroptosis initiates adaptive immune responses by releasing damage-associated molecular patterns (DAMPs) into the tissue microenvironment. After being phagocytosed, necroptotic cells can induce phagocytes (such as DCs and macrophages) to release proinflammatory cytokines, increase stimulatory molecules, and enhance cross-presentation, triggering a robust immune response [143]. Necroptotic cells can provide antigens and inflammatory cytokines to DCs for antigen cross-priming, activating cytotoxic CD8+ T lymphocytes [144]. Activated CD8+ T cells release multiple effector cytokines, exhibiting cytotoxic effects in vivo and avoiding tumor development. Although necroptosis plays a role in inducing and enhancing cancer immunity, evidence suggests that immune-inflammatory cells recruited by necroptosis/necrosis can promote tumor development by facilitating angiogenesis, promoting cancer cell proliferation, and accelerating cancer metastasis. Additionally, necroptotic/necrotic cells can release regulatory cytokines, such as IL-1α, which can directly stimulate the proliferation of neighboring cells and potentially promote tumor progression [145]. Activated inflammatory cells can also release reactive nitrogen intermediates (RNIs) and reactive oxygen species (ROS), which can damage DNA and lead to genomic instability, thereby promoting tumorigenesis [41].

#### 3.3.2. Tumor Metastasis

The role of necroptosis in metastasis exhibits duality. On the one hand, studies have shown that in osteosarcoma models, shikonin can significantly reduce primary tumors and lung metastases by inducing RIPK1- and RIPK3-dependent necroptosis. The antimetastatic effects of necroptosis may be related to its regulation of reactive oxygen species (ROS) production, which involves the role of necroptosis in the shedding and metabolism of the extracellular matrix (ECM), ultimately affecting cancer metastasis [146]. In fact, RIPK3 has been proven to regulate downstream ROS production and activate various metabolic enzymes to modulate TNF-induced ROS production. Together, these processes induce a large amount of ROS production during necroptosis, enabling necroptosis to kill metastatic cancer cells through ROS bursts. Therefore, necroptosis may be a key pathway for inhibiting tumor metastasis [41]. On the other hand, in certain circumstances, necroptosis may facilitate cancer cell metastasis. The process of tumor cells escaping from blood vessels and entering secondary sites (i.e., extravasation) is a critical step in metastasis. Studies have shown that endothelial cells co-cultured with tumor cells undergo necroptosis, and, similarly, mouse lung epithelial cells treated with metastatic tumor cells also exhibit necroptotic characteristics. Furthermore, the binding of DR6 to its ligand APP (amyloid precursor protein) promotes endothelial cell death and tumor cell extravasation. Endothelial cells undergoing necroptosis provide a pathway for tumor cells to begin extravasation, and/or DAMPs (damage-associated molecular patterns) released by necroptotic cells affect tumor cells and neighboring endothelial cells, thereby promoting tumor cell extravasation and metastasis. Therefore, therapies targeting DR6-mediated necroptosis in endothelial cells may represent a novel approach to preventing cancer metastasis [147].

### 3.4. Pyroptosis

An intricate and vital part of tumor growth is played by pyroptosis, an inflammatory kind of programmed cell death. The exact processes connecting pyroptosis to antitumor immunity are still unclear, despite the fact that a great deal of research has established its important involvement in cancer. There is evidence that pyroptosis can have both tumor-promoting and tumor-suppressing effects as the study of the processes of inflammatory cells in the tumor microenvironment continues [42].

#### 3.4.1. Tumor-Suppressing Effects

Research has progressively uncovered the mechanisms by which inflammatory cells operate within the tumor microenvironment. The development of ovarian cancer is closely associated with elevated levels of various inflammatory factors [148]. For instance, one study demonstrated that knocking down caspase-4 or GSDMD in ovarian cancer cells significantly reduced their cytotoxic activity, suggesting that a-NETA may modulate the pyroptosis pathway to exert its biological effects [43]. Another study found that nobiletin, a plant-derived compound from citrus fruits, can inhibit the malignant growth of ovarian cancer cells by regulating autophagy and inducing apoptosis and ROS-mediated pyroptosis [149]. Moreover, tumor cells undergoing pyroptosis can recruit immune cells that are typically suppressed by the tumor. A study using a bioorthogonal system revealed that pyroptosis in less than 15% of tumor cells in live animal models is sufficient to eliminate the entire tumor xenograft [150]. Researchers found that within the immune microenvironment created by pyroptosis, immune effector cells, such as CD8+ T cells and NK cells, can induce pyroptosis in tumor cells via granzyme B, establishing a positive feedback loop [151]. However, animals lacking cytotoxic cells or perforin exhibit impaired tumor suppression.

#### 3.4.2. Tumor-Promoting Effects

Upon activation, pyroptosis releases proinflammatory mediators, such as IL-1 and IL-18, which may drive the initiation and progression of cancer. Consequently, some researchers consider pyroptosis a tumor-promoting cell death mechanism akin to accidental necrosis. For instance, Gao et al. found that GSDMD protein levels are significantly higher in non-small cell lung cancer tissues compared to adjacent normal tissues. High GSDMD expression is associated with aggressive tumor features, including larger tumor size and advanced TNM stage [152]. However, recent studies have shown that exogenous activation of pyroptosis can significantly inhibit tumor growth. Various cancers in various systems have demonstrated sensitivity to pyroptosis induction [153]. Additionally, the distal HOXA gene transcript HOTTIP can increase AKT2 expression and inhibit the ASK1/JNK signaling pathway by negatively regulating mir-148a-3p. This, in turn, promotes ovarian cancer cell proliferation and NLRP1 inflammasome-mediated pyroptosis, ultimately accelerating the cancer’s malignant progression [154].

### 3.5. Ferroptosis

Ferroptosis is a novel form of regulated cell death, characterized by the iron-dependent accumulation of lipid ROS to lethal levels. Numerous studies have implicated ferroptosis in carcinogenesis. Strategies that manipulate the induction of ferroptosis have been shown to effectively suppress tumor development, including in some chemotherapy-resistant tumors [51]. The tumor suppressor p53 is closely associated with sensitivity to ferroptosis. In mice with intact p53, this protein binds to the promoter region of SLC7A11 and inhibits its transcription, which is essential for inducing ferroptosis. However, mice with mutations in multiple acetylation sites within p53 exhibit a marked loss of p53-dependent ferroptotic responses [155]. Activated CD8+ T cells from immunotherapy can induce ferroptosis in tumor cells in vivo. These T cells downregulate the expression of SLC7A11. The IFN-γ produced by CD8+ T cells increases the binding of STAT1 to the transcription start site of SLC7A11, thereby inhibiting its transcription. In tumor cells, the absence of STAT1 abolishes the IFN-γ-mediated downregulation of SLC7A11 and reverses RSL3-induced lipid peroxidation and cell death [156,157]. In contrast, tumor cells that are resistant to ferroptosis or treated with ferroptosis inhibitors are insensitive to PD-L1 inhibitor therapy. Further in vivo experiments have demonstrated that T cells can induce ferroptosis in mice bearing ovarian tumors. Immunohistochemical studies have revealed a negative correlation between CD8 levels and the expression of the Xc- complex, suggesting that sensitivity to ferroptosis is parallel to antitumor immunity [158]. Additionally, accumulating evidence indicates that the increased production of prostaglandin E2 (PGE2) within tumors facilitates immune evasion by cancer cells [159].

## 4. Conclusions

The complex relationship between regulated cell death (RCD) mechanisms and cancer underscores the importance of understanding the dual roles of autophagy, apoptosis, necroptosis, pyroptosis, and ferroptosis in tumor development and progression (Table 1). While autophagy and apoptosis primarily function as tumor suppressors by maintaining genomic stability and eliminating damaged cells, they can also promote cancer progression by supporting tumor cell survival, inducing drug resistance, and modulating the tumor microenvironment. Similarly, necroptosis, pyroptosis, and ferroptosis exhibit both tumor-suppressive and tumor-promoting effects, depending on the specific context. The crosstalk between these RCD mechanisms further complicates their roles in cancer. Therefore, future cancer therapies should consider the context-dependent nature of RCD mechanisms, aiming to selectively target their tumor-promoting functions while preserving their tumor-suppressive activities [160]. This approach holds promise for developing more effective and personalized cancer treatments.

## Figures and Tables

**Table 1:** Interactions between regulated cell death mechanisms in cancer.

Interaction Type	Description	Location
Autophagy and Apoptosis	Autophagy can degrade inhibitors of pro-apoptotic factors, indirectly activating apoptosis. p53 exerts dual roles in autophagy regulation based on its subcellular localization: cytoplasmic p53 inhibits autophagy via mTORC1 activation, while nuclear p53 activates autophagy via AMPK/mTOR.	Section 3.1.2
Autophagy and Apoptosis	Excessive autophagy can lead to cell death via over-degradation of critical components, which is a form of autophagic cell death. This process can inhibit tumor progression.	Section 3.1.2
Autophagy and Apoptosis	Autophagy can induce cell cycle arrest and senescence, which can suppress tumor proliferation. This is related to the regulation of the autophagy and apoptosis pathways.	Section 3.1.3
Autophagy and Inflammation (Necroptosis)	Autophagy can degrade inflammasomes and damaged lysosomes, preventing the release of proinflammatory cytokines and thus suppressing protumor inflammation. This is part of the “chronic inflammation–fibrosis–cancer” axis.	Section 3.1.4
Autophagy and Inflammation (Necroptosis)	Autophagy can clear damaged mitochondria and inflammasomes, which helps to prevent infection-related cancers. This is related to the degradation of inflammasomes by autophagy.	Section 3.1.4
Autophagy and Viral Carcinogenesis (Necroptosis)	Autophagy can degrade oncogenic viruses (e.g., HBV, HPV), but these viruses can evade clearance by encoding autophagy inhibitors. This interaction is important in viral carcinogenesis.	Section 3.1.4
Autophagy and Pyroptosis	Autophagy can negatively regulate pyroptosis by eliminating damaged mitochondria and inflammasome components. This interaction helps to control inflammatory responses.	Section 3.4
Autophagy and Ferroptosis	Autophagy can promote ferroptosis through ferritin degradation (ferritinophagy). This interaction can influence cellular sensitivity to ferroptosis.	Section 3.5
Apoptosis and Necroptosis	Caspase-8 can suppress necroptosis through RIPK1/RIPK3 cleavage while also executing apoptotic signaling. This dual functionality of caspase-8 links the two pathways.	Section 3.3 and Section 3.4
Necroptosis and Pyroptosis	Synergistic interactions between MLKL (necroptosis effector) and gasdermin D (pyroptosis effector) can amplify inflammatory responses. This interaction highlights the crosstalk between these two forms of cell death.	Section 3.3 and Section 3.4
Ferroptosis and Apoptosis	p53, a key regulator of apoptosis, also plays a dual role in ferroptosis regulation. Nuclear p53 can inhibit ferroptosis by upregulating SLC7A11, while under certain stress conditions, p53 may promote ferroptosis.	Section 3.5
Ferroptosis and Apoptosis	Anti-apoptotic Bcl-2 family proteins may influence ferroptosis progression by modulating mitochondrial function. This interaction shows the crosstalk between the apoptosis and ferroptosis pathways.	Section 3.5

Note: This table summarizes the key interactions between regulated cell death mechanisms (autophagy, apoptosis, necroptosis, pyroptosis, and ferroptosis) as described in this document. The interactions are categorized by the types of cell death involved, with brief descriptions and corresponding paragraph locations provided for reference.
